# Biological subphenotypes in patients hospitalized with suspected infection in Thailand: a secondary analysis of a prospective observational study

**DOI:** 10.1016/j.lansea.2025.100536

**Published:** 2025-01-30

**Authors:** Prapassorn Poolchanuan, Taylor D. Coston, Viriya Hantrakun, Parinya Chamnan, Gumphol Wongsuvan, Pavan K. Bhatraju, Narisara Chantratita, Direk Limmathurotsakul, T. Eoin West, Shelton W. Wright

**Affiliations:** a Department of Microbiology and Immunology, Faculty of Tropical Medicine, Mahidol University, Bangkok, Thailand; b Division of Pulmonary, Critical Care and Sleep Medicine, Department of Medicine, University of Washington, Seattle, WA, USA; c Mahidol-Oxford Tropical Medicine Research Unit, Faculty of Tropical Medicine, Mahidol University, Bangkok, Thailand; d Cardiometabolic Research Group, Department of Social Medicine, Sunpasitthiprasong Hospital, Ubon Ratchathani, Thailand; e Department of Tropical Hygiene, Faculty of Tropical Medicine, Mahidol University, Bangkok, Thailand; f Department of Global Health, University of Washington, Seattle, USA; g Division of Pediatric Critical Care Medicine, Department of Pediatrics, University of Washington, Seattle, WA, USA

**Keywords:** Global infection, Subphenotypes, Latent class analysis, Resource-limited, Tropical infection

## Abstract

**Background:**

Subphenotypes of infected patients have been reported in Europe and North America, but few studies have investigated populations in Southeast Asia. We sought to identify and differentiate subphenotypes of patients hospitalized with suspected infection in rural Thailand using biological markers implicated in the dysregulated host response.

**Methods:**

In a cohort of prospectively enrolled patients hospitalized with suspected infection in northeastern Thailand, we measured 15 circulating biomarkers from a random selection of 585 subjects and developed latent profile models to identify subphenotypes. Patient characteristics were compared after subphenotype assignment, and a parsimonious model was developed to identify patient subphenotypes.

**Findings:**

We identified and assigned 585 patients to three subphenotypes termed latent biological profile (LBP)-1 (52%), LBP-2 (39%) and LBP-3 (9%). Patients assigned to LBP-3 had a higher risk of 28-day mortality compared to those in LBP-1 and LBP-2 (adjusted relative risk 1.8, 95% confidence interval [CI] 1.1–2.9, P = 0.02). Patient clinical characteristics and biomarker concentrations also differed by subphenotype assignment. A parsimonious three-biomarker model identified subphenotypes in an internal validation cohort (LBP-1: area under the receiver operating curve [AUC] 0.96, 95% CI: 0.94–0.98; LBP-2: AUC 0.77, 95% CI 0.71–0.83; LBP-3: AUC 0.99, 95% CI 0.98–1.00).

**Interpretation:**

We identified three biological subphenotypes of patients with suspected infection in rural Thailand, where the burden of infection is high but understudied. Patient subphenotype assignment was characterized by distinct clinical outcomes and biological profiles which could inform contextualized future study design.

**Funding:**

The US 10.13039/100000002National Institutes of Health, the 10.13039/100010269Wellcome Trust, and the Firland Foundation.

**Research in context**

**Evidence before this study**

As part of the emergence of precision medicine, categorizing patients with heterogenous diseases into specific subgroups has rapidly developed in resource-rich areas, including Europe and North America. With a goal of identifying specific subgroups of patients who may benefit from targeted therapeutics, patients with infection-related diseases, including sepsis and acute respiratory distress syndrome (ARDS), have been increasingly categorized into subphenotypes. However, few such studies have examined patients in tropical or resource-constrained regions, where etiologies of infection and management may differ. We searched PubMed for published articles with a combination of “infection” and “subphenotype”, prior to September 2024, identifying 260 studies. Several identified studies assessed both clinical and biological subphenotypes in hospitalized patients with sepsis and ARDS, with identified differential responses by subphenotype to clinical trial interventions. No identified study included patients in Southeast Asia, and a single study included patients in a rural setting in Africa.

**Added value of this study**

This is the largest study in a low- or middle-income setting to identify subphenotypes in patients with suspected infection. Additionally, this is the only reported study to identify infection-related biological subphenotypes in patients in Southeast Asia. Using a cohort of prospectively enrolled patients hospitalized with suspected infection in rural Thailand, we identified three distinct subphenotypes with differing patient mortality. Additionally, patients assigned to each subphenotype differed in their biological profiles and infectious etiologies suggesting potentially distinct pathophysiologic processes. Finally, we report a novel panel combining three biomarkers of innate immune inflammation and endothelial dysfunction that can identify these subphenotypes. Our study's strengths include its rigorous data collection and statistical approach. Furthermore, as in-hospital mortality assessments might underestimate mortality outcomes in low- and middle-income settings where patients may prefer to die in their homes, we followed all patients to 28 days after admission, including after discharge.

**Implications of all the available evidence**

Our findings indicate that patients hospitalized with suspected infection in rural Southeast Asia can be categorized into distinct, biologically derived subphenotypes. These results extend the existing literature reporting subgroups of patients with infection-related diseases in resource-rich areas, but also suggest potentially unique subphenotypes exist in resource-limited settings. Given the burden of sepsis globally, identifying patients who will benefit most from specific interventions is an urgent priority. Therefore, categorizing patients by subphenotype could have immediate implications in patient triage and prognosis but could also provide the necessary basis for patient-specific, contextualized interventional trials.

## Introduction

Sepsis is a leading cause of death worldwide and is characterized by organ dysfunction due to a dysregulated immune response during infection.^1^ Although the global burden of sepsis is highest in resource-constrained areas, it remains understudied in these regions.^2^ Additionally, critical care capacity is often limited in resource-constrained settings causing many patients with sepsis to be treated outside of a traditional intensive care unit (ICU).^3^ Importantly, infection-related outcomes in ICU patients are worse in low-or-middle income countries (LMICs) compared to high-income countries-including in Asia-even when accounting for severity of illness and critical care management strategies.^4^

Recently, the concept of precision medicine has emerged with a goal towards identifying interventions with the highest likelihood of success for groups of patients with specific, shared characteristics.^5^ Multiple recent studies of patients with sepsis-related diseases have identified distinct subgroups, also termed subphenotypes, with variations in their response to management.^6^^,^^7^ High quality studies categorizing patients with suspected infection in LMICs—where most global cases of sepsis occur—are limited.^8^ In resource-limited regions of Southeast Asia, infection-related hospitalization is common, and host factors and etiologic pathogens are distinctive from other parts of the world.^9^ Therefore, further context-specific research is urgently needed.

Phenotyping infected patients frequently involves identifying groups of patients with shared characteristics.^7^ Two frequently-used mixture model approaches include latent class analysis (using categorical ± continuous indicator variables) and latent profile analysis (using only continuous indicator variables).^10^ Critically, infection-related subphenotypes have limited reproducibility across different populations, further supporting the need for contextualized research.^11^ In this study, we hypothesized that a set of circulating biomarkers from different infection-influenced systems could categorize adults hospitalized with suspected infection in rural Thailand into specific subphenotypes. This cohort was chosen for analysis because it represents an under-researched set of patients with wide regional representation and diverse infectious etiologies common to Southeast Asia.

## Methods

### Study design and participants

The primary Ubon-sepsis cohort has been described previously.^12^ In brief, subjects aged 18 years or older who were admitted with suspected infection to Sunpasittiprasong Hospital in Ubon Ratchathani, Thailand were prospectively enrolled within 24 h of admission between 2013 through 2017. Screening for recruitment occurred through examination of medical records of patients admitted to the hospital with suspected infection. Per hospital policy, all patients were first admitted to the emergency department and then subsequently transferred to the medical wards and medical ICUs. Patients admitted to the medical wards or medical ICU were eligible for enrollment. Subjects were enrolled within 24 h of study hospital presentation if they met three or more systemic manifestations of infection, as proposed by the 2012 Surviving Sepsis Campaign. At enrollment, a venous whole blood lactate was measured using a point-of-care device (Lactate Pro 2, Arkray, Australia). Additional clinical and laboratory data was obtained from the patient's medical records. After enrollment, patients were treated according to local standard of care and followed until 28-days after enrollment.

### Clinical definitions

A modified sequential organ failure assessment (modified SOFA) score was calculated for all subjects at the time of enrollment, given the absence of some data points such as inotrope and vasopressor agent doses and partial pressure of oxygen in arterial blood (PaO_2)._ Therefore, in the cardiovascular component of the SOFA score, 2/4 points were given for only dobutamine or dopamine infusions and 3/4 points were given for epinephrine or norepinephrine infusions. For the respiratory component of the SOFA score, 2/4 points were given if advanced respiratory support (endotracheal tube or mechanical ventilation) was utilized but arterial blood gas results were not available. This modified SOFA score has been previously described.^12^

### Biomarker selection

In this study, our goal was to use quantifiable biological variables to categorize patients with suspected infection, in part to aid in identifying molecular trends and to limit the influence of organ failure and severity of illness on clinical variables. We and others have identified several key biomarkers characterizing the host response to infection in rural Southeast Asia and similar regions.^8^^,^^13^, ^14^, ^15^ We therefore selected 14 of these previously identified biomarkers, with readily available testing in a resource-constrained setting, and categorized them into separate pathophysiologic domains: endothelial function (angiopoietin 1 [Ang-1], angiopoietin 2 [Ang-2], soluble fms-like tyrosine kinase 1 [sFlt-1]); coagulation (plasminogen activator inhibitor-1 [PAI-1], platelet count); cytokine release (tumor necrosis factor [TNF], interleukin 6 [IL-6], interleukin 8 [IL-8], interleukin 1 beta [IL-1β], interleukin 10 [IL-10]); innate immune systemic inflammation (soluble triggering receptor expressed by myeloid cells 1 [sTREM-1], soluble tumor necrosis factor receptor 1 [sTNFR-1], white blood cell count [WBC] and neutrophil percentage). Additionally, serum glucose at the time of enrollment was included as a metabolic biomarker frequently altered during infection. Although appropriate sample size for latent profile analysis has not been clearly established, sample sizes >500 are recommended for improved accuracy.^10^ From the primary cohort of 4989 with 28-day outcome data, a sample of 585 subjects, given the availability of electrochemiluminescence reagents, was randomly selected for analysis from the primary cohort using the *sample* command in Stata. Plasma samples were available for all 585 subjects selected.

### Biomarker assays

Biomarker assays were performed on plasma samples obtained at the time of enrollment in the analysis cohort. The concentrations of Ang-1, Ang-2, sFlt-1, TNF, IL-6, IL-8, IL-1β, IL-10, sTREM-1, PAI-1, and sTNFR-1 were measured by electrochemiluminescence multiplex assay (Meso Scale Discovery, Rockville, MD). A subset of these biomarkers (IL-8, sTREM-1, and Ang-1) was previously described as part of a larger cohort.^13^ Samples were diluted based on the sensitivity of the assays, and upper and lower limits of detection were determined by the manufacturer's software for each plate. For sample concentrations below the lower limit of detection, a random value between the lower limit of detection and one half the lower limit of detection was assigned to maintain variability. Positive and negative plasma control samples were used across electrochemiluminescence plates, including to assess plate variability. Additionally, plasma samples were assessed consecutively by study enrollment, and the lack of inter-plate biomarker concentration variability was assessed prior to downstream analysis.

### Laboratory data

Data regarding subject white blood cell count (WBC), percent neutrophils, platelet count and serum glucose obtained on the day of enrollment was obtained from the study hospital's clinical laboratory.

### Statistical analysis

#### Latent profile analysis

The 15 *a priori*-selected biomarkers were considered as class-defining variables. Right-skewed biomarkers were log_10_-transformed and left-skewed biomarkers were reflected then log_10_-transformed. Next, standardized z-scores were calculated for each variable. A correlation matrix was then constructed and used to assess pairwise correlation between variables. Although variable correlation may affect latent class and latent profile analyses differently, broadly accepted cutoffs are debated and range from 0.5 to 0.75 in similar studies.^10^^,^^16^^,^^17^ Therefore, an *a priori* decision was made to identify biomarkers with high correlation (coefficient >0.7) and assess for removal from the latent profile analysis.

Subsequently, a latent profile analysis (LPA) was performed on the remaining biomarkers for subphenotype classification, without consideration of clinical outcomes. Although missing biomarker data was minimal, LPA included the full-information maximum likelihood approach to missing data, estimating model parameters using both complete and incomplete data, per recommended guidelines.^10^^,^^18^ Four latent profile models were generated ranging from 2 to 4 subgroups. Final subgroup selection utilized multiple criteria, with the Vuong-Lo-Mendell-Rubin (VLMR) likelihood ratio test as the primary assessment for model fit.^19^ We also employed additional criteria: the Bayesian Information Criteria, the entropy statistic and unique profile interpretability.^20^ Sensitivity analyses were performed by developing LPA models including 1) all biomarkers without removal of those highly correlated and 2) removal of correlated biomarkers (coefficient >0.5). After a final LPA model was selected, patients were assigned to a respective subphenotype–termed Latent Biological Profile (LBP)–based on the highest probability of profile assignment (minimum >0.5).

### Subphenotype comparison

Poisson regression models were then developed to assess relative risk of 28-day mortality by subphenotype, both unadjusted and adjusting for age, sex, Charlson Comorbidity index, referral from another facility, modified SOFA score, and lactate concentration at the time of enrollment. Additionally, Kaplan–Meier survival curves were generated by subphenotype and comparisons made by the logrank test. Enrollment clinical data, including calculated clinical scores, were summarized using proportions for discrete variables and medians and interquartile ranges (IQR) for continuous variables. Differences in variables between groups were evaluated by the chi-square test or Kruskal–Wallis test.

### Parsimonious subphenotype classification model development

To assess whether a parsimonious number of variables could accurately classify subphenotype assignment, a derivation cohort was randomly selected consisting of 60% of the analysis cohort (N = 351). All 14 biomarkers used in the LPA were subjected to multinomial logistic regression analysis for LBP assignment by least absolute shrinkage and selection operator (LASSO) for subjects in the derivation cohort.^21^^,^^22^ For the multinomial LASSO regression model development and assessments, missing biomarker data was handled by using κ nearest neighbor imputation.^23^ A LASSO-selected biomarker model was then assessed for discrimination of LBP classification by multinomial logistic regression in the derivation cohort as well as an internal validation cohort of the remaining 40% of the analysis cohort (N = 234); model performance was assessed by receiver operating characteristic curve. Additional details, including model assessment, are included in the supplementary methods.

### Identification of previously identified subphenotypes

Subphenotypes developed in patients with acute respiratory distress syndrome (ARDS) have been validated in patients with sepsis in resource-rich settings.^7^^,^^24^^,^^25^ Therefore, we classified patients into ARDS subphenotypes (termed ARDS “hyperinflammatory” and “hypoinflammatory”) by applying a previously published parsimonious model including plasma concentrations of IL-8, sTNFR-1 and need of a vasopressor at enrollment (see supplementary methods).^24^

Latent profile analysis was performed using Mplus version 8.9 (Muthen and Muthen, Los Angeles, CA). All other analyses were performed using Prism (Graphpad. Boston, MA), Stata/SE version 14.2 (College Station, TX) or R statistical environment version 4.4.2 (R Foundation for Statistical Computing, Vienna, Austria).

### Ethics approval and consent to participate

Written informed consent was obtained from study participants or their representatives prior to enrollment. The studies were approved by the Sunpasitthiprasong Hospital Ethics Committee (039/2556), the Ethics Committee of the Faculty of Tropical Medicine, Mahidol University (MUTM2012-024-01 and MUTM2024-022-01), the University of Washington Institutional Review Board (42988) and the Oxford University Tropical Research Ethics Committee (OXTREC172-12 and 530–24).

### Role of the funding source

The study sponsors were not involved in the study design, data collection, analysis, interpretation, writing or decision to submit the manuscript for publication.

## Results

### Patient characteristics

Of the 4989 subjects enrolled into the original cohort with outcome data, 585 were randomly selected for biomarker measurement and analysis (Supplementary Figure S1). Descriptive characteristics are listed in Table 1. Fifteen percent of this selected set of patients died by 28-days after enrollment (86/585). Of the 585 patients analyzed, 288/585 (49%) were referred from another hospital in the region, and a total of 39 originating hospitals were represented. Infectious etiologies and presentation are provided in Supplementary Table S1. Of the cohort, 89 (15%) had a blood stream infection, 36 (6%) were diagnosed with dengue and 166 (28%) presented with a lower respiratory tract infection.

**Table 1:** Patient characteristics.

Characteristics	Analysis cohort (N = 585)	Original cohort (N = 4989)
**Demographics**		
Age in years, median (IQR)	59 (39–73)	57 (41–71)
Female sex, N (%)	283 (48)	2330 (47)
**Pre-existing conditions**		
Charlson comorbidity index, median (IQR)	2 (0–4)	2 (0–4)
Diabetes, N (%)	120 (21)	1006 (20)
Chronic liver disease, N (%)	15 (3)	133 (3)
Chronic kidney disease, N (%)	65 (11)	545 (11)
Chronic cardiovascular disease, N (%)	38 (7)	282 (6)
Chronic lung disease, N (%)	43 (7)	394 (8)
Cancer, N (%)	15 (3)	82 (2)
HIV, N (%)	6 (1)	63 (1)
**Duration of symptoms (days), median (IQR)**	2 (1–4)	3 (1–4)
**Lactate (mmol/L), median (IQR)**	1.8 (1.3–2.8)	1.8 (1.2–2.8)
Lactate ≥2, N (%)	253 (43)	2141 (43)
**Modified SOFA score, median (IQR)**	3 (1–5)	4 (2–6)
Modified SOFA ≥2, N (%)	400 (69)	3806 (76)
**28-day mortality, N (%)**	86 (15)	819 (16)

### Identification of biomarker-based subphenotypes

A set of 15 biomarker variables were available to develop the latent profile models. Biomarkers had minimal data missing as detailed in Supplementary Table S2. A correlation matrix demonstrated high correlation (>0.7) between sTFNR-1 and both sTREM-1 and TNF and so sTNFR-1 was removed as a biomarker variable for the LPA (Supplementary Table S3). In the LPA of the remaining 14 biomarker variables, a three-profile model was selected as it had a lower log likelihood, BIC and entropy compared to a two-profile model, and a four-profile model did not provide significantly better fit based on the VLMR test. LPA model fit characteristics and probability of profile assignment are detailed in Supplementary Table S4.

A sensitivity analysis was then performed generating an LPA model retaining all 15 variables, including sTNFR-1. This larger biomarker model also favored a three-profile model (Supplementary Table S5). An additional sensitivity analysis was performed without biomarkers with correlation coefficients >0.5, removing sTNFR-1, IL-6, IL-8, TNF and sTREM-1. The subsequent LPA of the 10 remaining biomarker variables again favored a three-profile model (Supplementary Table S6). Due to the specific loss of potentially biologically relevant variables related to both cytokine release and innate-immune systemic inflammation in this reduced model and considerations of correlation in LPA, the primary 14-biomarker LPA was used to assign patients to subphenotypes, termed Latent Biological Profiles (LBP) 1–3.

### Subphenotypes characteristics

**Fig. 1:**
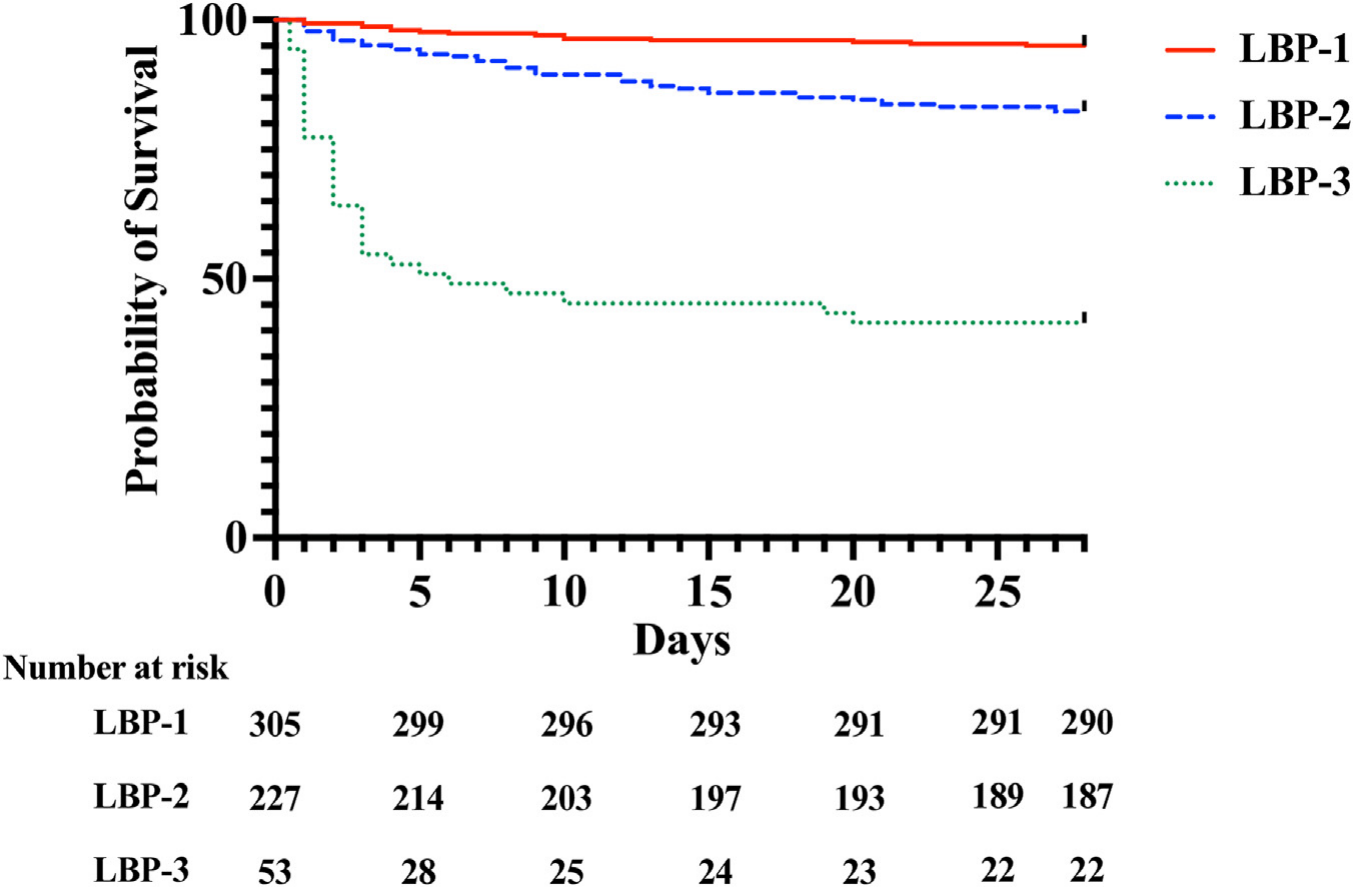
**Survival by subphenotype**. Kaplan–Meier survival curves over 28-days for each latent biological profile (LBP; P < 0.001; logrank test).

All patients were assigned to an LBP subphenotype. The characteristics of patients assigned to each LBP are listed in Table 2. Patient referral from another facility differed by subphenotype assignment (P < 0.001), though patients did not differ in time to transfer (median time <1 day from presentation, all; P = 0.92) nor duration of symptoms (median 2–3 days, all; P = 0.08). Subphenotype patients did differ by lactate concentration, modified SOFA score, and critical care interventions at enrollment (P < 0.001, all). Twenty-eight-day mortality varied by subphenotype assignment (P < 0.001): LBP-1: 5% (15/305), LBP-2: 18% (40/227), and LBP-3: 59% (31/53). Patient survival by subphenotype assignment is illustrated in Fig. 1. In patients with modified SOFA ≥2, many baseline patient characteristics were similar between assigned LBP, but differences were again noted in lactate concentration, modified SOFA score, critical care interventions and 28-day mortality (P < 0.001, all; Supplementary Table S7).

**Table 2:** Characteristics and outcome based on subphenotype.

Characteristics	LBP-1 (N = 305)	LBP-2 (N = 227)	LBP-3 (N = 53)	P-value
**Demographics**				
Age in years, median (IQR)	55 (33–73)	63 (50–74)	63 (47–73)	<0.001
Female sex, N (%)	170 (56)	93 (41)	20 (38)	0.001
**Pre-existing conditions**				
Charlson Comorbidity Index, median (IQR)	1 (0–4)	3 (1–4)	2 (1–4)	<0.001
Diabetes, N (%)	56 (18)	56 (25)	8 (15)	0.12
Liver disease, N (%)	7 (2)	5 (2)	3 (6)	0.33
Kidney disease, N (%)	19 (6)	37 (16)	9 (17)	<0.001
Cardiovascular disease, N (%)	19 (6)	16 (7)	3 (6)	0.9
Lung disease, N (%)	25 (8)	15 (7)	3 (6)	0.70
Cancer, N (%)	9 (3)	5 (2)	1 (2)	0.82
HIV, N (%)	3 (1)	1 (0.4)	2 (4)	0.10
**Duration of symptoms**, median (IQR)	2 (1–3)	3 (1–4)	3 (1–3)	0.08
**Referred**, N (%)	116 (38)	135 (60)	37 (70)	<0.001
Days to transfer, median (IQR)	0 (0–0)	0 (0–0)	0 (0–0)	0.92
**Lactate** (mmol/L)	1.4 (1.1–1.9)	2.1 (1.6–3.1)	6.4 (4.2–9.2)	<0.001
**Modified SOFA score**, median (IQR)	1 (0–3)	4 (2–6)	9 (6–12)	<0.001
**Critical care at enrollment**				
Mechanical ventilation, N (%)	26 (9)	45 (20)	29 (55)	<0.001
Receiving vasoactive medications, N (%)	31 (10)	56 (25)	31 (59)	<0.001
ICU admission, N (%)	7 (2)	14 (6)	15 (28)	<0.001
**28-day mortality**, N (%)	15 (5)	40 (18)	31 (59)	<0.001
Days to death^a^, median (IQR)	6 (3–13)	8 (3–15)	2 (1–3)	<0.001

Relative risk of death	Unadjusted^b^ (95% CI)	P-value	Adjusted^c^ (95% CI)	P-value
LBP-1 vs. LBP-2/3	0.2 (0.1–0.3)	<0.001	0.4 (0.2–0.8)	<0.001
LBP-2 vs. LBP-1/3	1.4 (1.0–2.0)	0.11	1.2 (0.8–1.7)	0.45
LBP-3 vs. LBP-1/2	5.7 (4.0–7.9)	<0.001	1.8 (1.1–2.9)	0.02

a Days between initial healthcare facility presentation (including referring facility) and death.

b Relative risk estimates for LBP assignment and 28-day mortality.

c Relative risk estimates for LBP assignment and 28-day mortality adjusting for age, sex, Charlson Comorbidity index, referral from another facility, modified SOFA score, and lactate concentration at the time of enrollment.

In regression models of the entire analysis cohort, assignment to LBP-3 was associated with an increased risk of 28-day mortality compared to LBP-1 or LBP-2 assignment in both an unadjusted model and a model adjusting for age, sex, pre-existing conditions, referral from another facility, modified SOFA score, and enrollment venous lactate concentration (adjusted relative risk 1.8, 95% confidence interval [95% CI] 1.1–2.9, P = 0.02; Table 2).

**Fig. 2:**
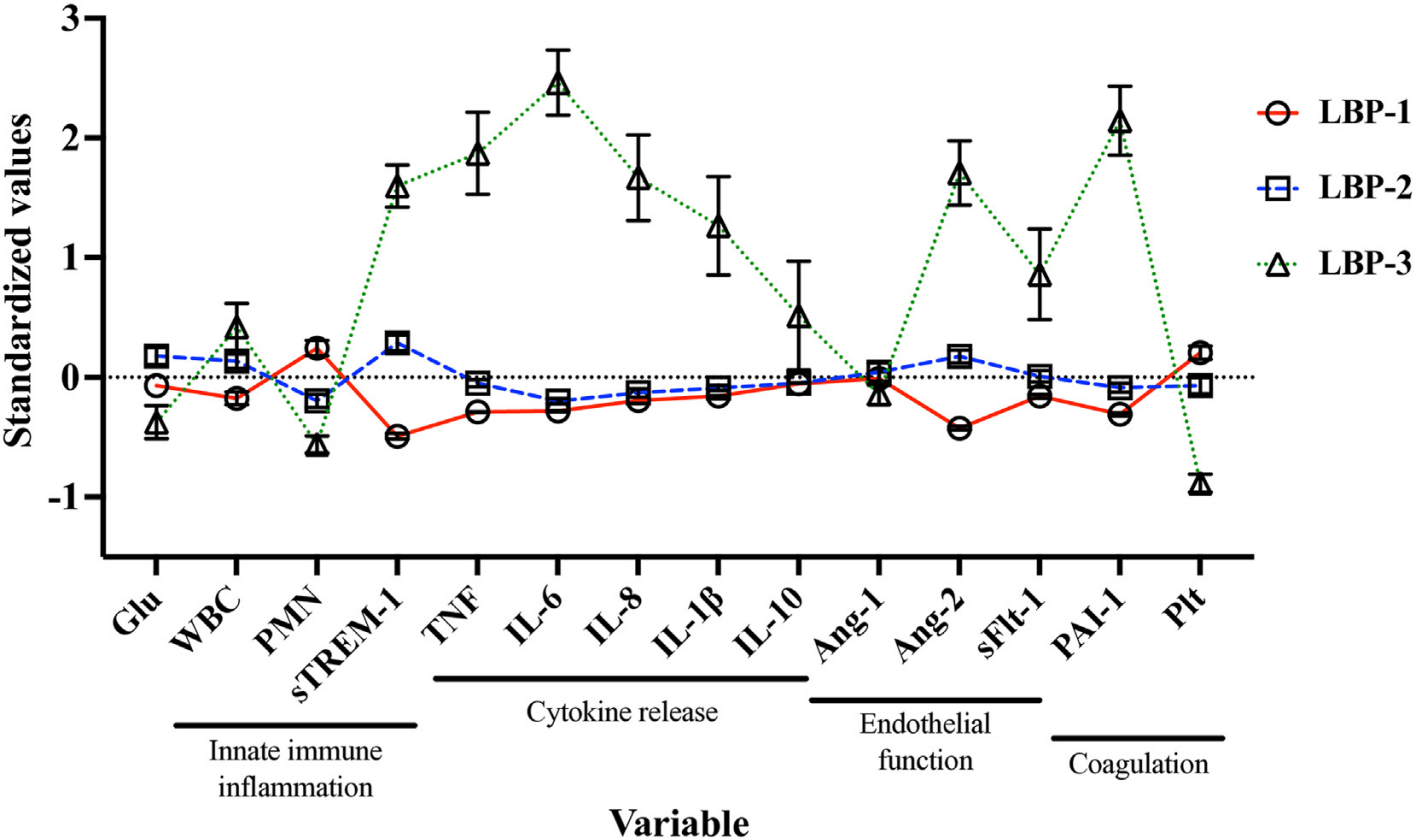
**Biological variables by subphenotype**. Standardized values (z-score) for all variables included in the primary model to assign subphenotype. Variables are sorted based on biological category. Mean and standard error of the mean are presented. Plt, platelet count; Glu, glucose; Ang, angiopoietin; WBC, white blood cell count; IL, interleukin; PMN, neutrophil percentage; sTREM-1, soluble triggering receptor expressed by myeloid cells 1; PAI-1, plasminogen activator inhibitor 1; TNF, tumor necrosis factor.

Infectious etiology frequently differed by subphenotype assignment. Gram-negative and Gram-positive blood stream infections were common in patients assigned to LBP-3 (26% and 13%, respectively; Supplementary Table S8). Of the patients assigned to LBP-1, 31/305 (10%) were diagnosed with dengue compared with no patients in LBP-3. Additionally, all patient biomarker concentrations differed across subphenotypes (all P = 0.001), apart from Ang-1 (P = 0.90; Fig. 2, Supplementary Table S9). Patients assigned to LBP-3 tended to have the highest concentrations of cytokines and markers of endothelial dysfunction.

### Subphenotype classification

We next sought to determine if a parsimonious biomarker panel could accurately classify LBP subphenotypes. In a derivation cohort comprised of 60% of the primary analysis cohort (N = 351), IL-6, sTREM-1 and Ang-2 were the top variables selected by multinomial LASSO regression for subphenotype classification. This three-biomarker model yielded the following area under the receiver operating characteristic curves (AUC) for subphenotype assignment: LBP-1: 0.97 (95% CI 0.96–0.98); LBP-2: 0.78 (95% CI 0.73–0.83) and LBP-3: 0.99 (95% CI 0.98–1.00; Table 3, Supplementary Table S10A, Supplementary Figure S2A–C). Reduced models including IL-6 ± sTREM-1 (the order in which biomarkers were selected in the LASSO regression) demonstrated strong but reduced performance (Supplementary Table S10B and C). In an internal validation cohort (N = 234), the three-biomarker (IL-6, sTREM-1 and Ang-2) model yielded the following AUC's for subphenotype assignment: LBP-1: 0.96 (95% CI 0.94–0.98), LBP-2: 0.77 (95% CI 0.71–0.83) and LBP-3: 0.99 (95% CI 0.99–1.00; Table 3, Supplementary Table S11A, Supplementary Figure S2D–F). Compared to reduced IL-6 ± sTREM-1 models, the three-biomarker model had the highest accuracy (0.86) and weighted F1-score (0.89) in the internal validation cohort (Supplementary Table S11).

**Table 3:** Parsimonious biomarker model for subphenotype classification.

IL-6 sTREM-1 Ang-2	Derivation cohort (N = 351)			Internal validation cohort (N = 234)		
	AUC (95% CI)	Sensitivity	Specificity	AUC (95% CI)	Sensitivity	Specificity
LBP-1	0.97 (0.96–0.98)	0.88	0.94	0.96 (0.94–0.98)	0.87	0.93
LBP-2	0.78 (0.73–0.83)	0.86	0.64	0.77 (0.71–0.83)	0.85	0.59
LBP-3	0.99 (0.98–1.00)	0.97	0.93	0.99 (0.98–1.00)	1.00	0.93

Model performance based on Youden index threshold which maximizes sensitivity and specificity.

### Concordance with previously identified subphenotypes

We next identified ARDS subphenotypes (previously termed “hyperinflammatory” and “hypoinflammatory”) derived in North America and validated in patients with sepsis.^7^^,^^24^ In the entire cohort of patients with suspected infection, 355/585 (61%) were classified as hyperinflammatory and 230/585 (39%) were classified as hypoinflammatory (Supplementary Table S12). When restricting the cohort to patients with SOFA ≥2, most patients were again classified as hyperinflammatory (311/400, 78%), a trend inconsistent with prior reports.^24^^,^^25^ Patients assigned to the hyperinflammatory subphenotype had higher 28-day mortality compared to those assigned to the hypoinflammatory subphenotype in both the entire cohort (22% vs. 4%, P < 0.001) and when restricted to those with SOFA ≥2 (24% vs. 9%, P < 0.01). The hyperinflammatory subphenotype represented patients assigned to all three LBP subphenotypes and did not differentiate between patients assigned to the LBP-2 and LBP-3 subphenotypes (Supplementary Table S12, Supplementary Figure S3). Additionally, 27% (95/355) of the patients in the hyperinflammatory subphenotype were also assigned to the LBP-1 subphenotype. Even when restricting to those with SOFA ≥2, 24% (76/311) of patients assigned to the hyperinflammatory subphenotype were also assigned to the LBP-1 subphenotype.

## Discussion

In this study, we identify three subphenotypes derived from biomarkers of patients hospitalized with suspected infection in northeastern Thailand, with correspondingly different mortality risks. We also report that a parsimonious three-biomarker panel, combining an inflammatory cytokine (IL-6), a marker of innate immune activation (sTREM-1) and endothelial dysfunction (Ang-2), can accurately identify patient subphenotype. These potentially novel subphenotypes could help inform future study design in resource-constrained areas of Southeast Asia, where the burden of infection and sepsis is high.

Few studies have sought to identify subphenotypes of infected patients in resource-limited areas despite the clinical promise of these approaches.^26^ One study, in Uganda, revealed three subgroups of predominantly HIV-infected sepsis patients which differed in outcomes.^8^ In that report, patients with relatively higher concentrations of certain biomarkers, including sTREM-1, Ang-2 and sFlt-1, were classified together and had worse outcomes, perhaps reflecting those patients classified into the LBP-3 subphenotype in our study. Furthermore, the reported distinct subphenotype highlighted by low Ang-1 concentrations was not reflected in our study, where subphenotypes did not differ by Ang-1 concentration.

In high-resource settings, several subphenotyping studies of infected patients have focused on the critically ill, typically meeting definitions of sepsis or ARDS.^25^^,^^27^, ^28^, ^29^ Additionally, in these reports subphenotypes are frequently derived from different combinations of clinical variables, hospital laboratory data and specifically measured biomarkers, and do not identify comparable populations.^25^ A widely applied approach has categorized patients into either hypo- or hyperinflammatory subphenotypes, with the hyperinflammatory group typically characterized by smaller size and higher mortality.^7^^,^^24^^,^^25^^,^^27^^,^^30^ Therefore, when applying this approach to our cohort composed of patients with and without sepsis and lower overall mortality, we expected a relatively small hyperinflammatory group. On the contrary, in our cohort, the hyperinflammatory subphenotype was much larger than the hypoinflammatory subphenotype, particularly after restricting to those meeting sepsis criteria. Therefore, the locally derived LBP subphenotypes may provide more subtle categorization of infected patients, particularly those at highest risk. For example, the hyperinflammatory subphenotype encompassed most of the LBP-2 and LBP-3 subphenotypes and nearly one-third of the LBP-1 subphenotype. While the LBP-3 subphenotype may represent a “hyperinflammatory” state, the biological processes underlying the LBP-1 and LBP-2 subphenotypes may be more nuanced. Indeed, our identification of the LBP-2 subphenotype, characterized by specific increases in sTREM-1 and Ang-2, appears to be distinct in comparison to previously reported subphenotypes in resource-rich settings.^7^ Additionally, in terms of outcome risk, the LBP subphenotypes may also have the additional advantage of identifying a larger “lower risk” group in LBP-1 and a particularly “high risk” group in LBP-3. Taken together, these results suggest existing subphenotypes may have different application in resource-constrained settings and discordance with locally derived subphenotypes.

An intrinsic challenge in categorizing patients with infected-related illnesses is whether severity of illness serves as an underlying latent variable.^31^ As sepsis is, in part, currently defined by organ failure and increased risk of death, severity of illness may be difficult to separate from infection-related subgroups.^32^ We intentionally included patients with suspected infection rather than restricting to current sepsis criteria, as these may not be applicable in many resource-limited settings.^3^ We also did not include clinical variables which may be markers of organ failure. Nonetheless, the biomarkers chosen for this study likely are influenced by or directly impact the development of organ failure. Nevertheless, after adjusting for a modified SOFA score, lactate and other potential confounding variables, patient subphenotype assignment was still associated with a differing risk of death, including in patients meeting current sepsis criteria. These findings suggest severity of illness does not completely explain subphenotype assignment.

How to best translate subphenotypes into current clinical practice remains a topic of debate.^33^ Subphenotype identification holds promise as a foundation of future precision medicine, including revealing targets for both point-of-care assays as well as novel therapeutics. However, further study of our identified phenotypes is necessary to determine whether underlying mechanistic differences in host responses may determine subphenotype assignment. Nevertheless, the inclusion of underrepresented, tropical infectious etiologies in our cohort is also unique. For example, patients with dengue were overrepresented in the LBP-1 subphenotype while melioidosis was overrepresented in the LBP-3 subphenotype. Whether these subphenotype differences represent unique host responses to these pathogens is unknown and further investigation is needed to determine if subphenotype assignment could also provide diagnostic or prognostic information for clinicians.

A parsimonious biomarker model including IL-6, sTREM-1 and Ang-2 had high accuracy to identify the LBP-1 and LBP-3 subphenotypes. The added complexity of accurately classifying three subphenotypes by a single model may be reflected in the decreased performance for LBP-2 assignment. Notably, IL-6 with and without sTREM-1 also had strong accuracy of correctly classifying LBP-1 & LBP-3 subphenotypes and could be used on its own for their accurate identification. Patients assigned to LBP-2 were characterized by specific elevations in sTREM-1 and Ang-2 compared to those in the LBP-1 subphenotype, with correspondingly higher mortality. Triggering receptor expressed on myeloid cells-1 (TREM-1) is expressed on innate immune cells and its activation may intensify inflammatory responses.^34^ sTREM-1, the soluble biomarker, has been associated with sepsis mortality, including in SE Asia.^13^^,^^35^^,^^36^ A recent trial of a TREM-1 inhibitor in patients with septic shock did not improve SOFA scores but subgroup analyses suggested potential efficacy in patients with higher sTREM-1 concentrations.^37^ Similarly, Ang-2 is an important regulator of endothelial activation, potentially leading to endothelial instability and facilitating microvascular obstruction.^38^ Ang-2 concentrations can predict poor sepsis-related outcomes, and therapeutics targeting the Ang-2 pathway are being investigated pre-clinically.^13^^,^^39^ While certain subphenotypes may benefit from targeted therapeutics, it is unknown if our reported subphenotypes represent distinct biological processes or rather clusters of patients on a spectrum.

Our study has several strengths. As far as we are aware, this is the largest study to identify biomarker-based subphenotypes of adults with suspected infection in a resource-limited setting. While this is a single-center study, the referral catchment area is large, and 39 originating hospitals were represented in the analysis cohort. Additionally, we employed broadly accepted latent profile analysis approaches. Our dataset featured limited missing data, near-universal follow-up, and we analyzed only variables from within the first 24 h after hospital admission. We also developed a three-biomarker profile classification model to facilitate application.

Our study also has several important limitations. Our biomarker selection process was not unbiased and other approaches might categorize patients differently. Reflecting the resource-constrained setting, our study was limited to a single site, required modification of the SOFA score and an appropriate external validation cohort was not available. Additionally, many patients were referred from another facility, though the vast majority were transferred within hours, and patients did not differ in time from initial presentation. However, subphenotype assigned patients differed by frequency of referral and clinician assessment of need for referral may contribute to subphenotype assignment. We also do not know if patients assigned to the three subphenotypes differ in their responses to management interventions. Finally, to measure biomarkers, we used a highly sensitive electrochemiluminescence assay which may not be available in resource-constrained areas. Future study of alternative methods to measure protein concentration, such as ELISAs, would facilitate implementation strategies for biomarker-based subphenotype identification.

In conclusion, we have identified three distinct biomarker-based subphenotypes of patients with suspected infection in rural Southeast Asia, a region where the infection burden is high but understudied. These subphenotypes demonstrate differences in clinical outcomes and could provide the foundation for understanding unique sepsis pathophysiology or identifying targets for intervention.

## Contributors

P.P. and S.W.W participated in project design, performed biomarker measurements, and analyzed the data. T.D.C. analyzed the data and participated in project design. V.H. oversaw sample and data collection and management. P.C. and G.W. participated in project design and facilitated sample and data collection. N.C. & P.K.B. participated in study and analysis design. D.L. and T.E.W. directed the original cohort study. T.E.W. and S.W.W. developed the project and study design and oversaw the study. S.W.W and T.D.C. accessed and verified the underlying data reported in the manuscript. All authors contributed to the writing or revision of the manuscript.

## Data sharing statement

The dataset supporting these findings are available in a repository upon publication (10.6084/m9.figshare.25537690).

## Declaration of interests

This work was supported by the US National Institutes of Health (grant numbers T32HL007287, F32HL168809, K08HL157562, R01HL113382 and R01AI137111). This research was also funded, in part, by the Wellcome Trust (090219/Z/09/Z and 101103/Z/13/Z). Finally, this research was supported by a Firland Foundation Grant and a University of Washington Global Innovation Fund grant.

A CC BY or equivalent license is applied to the authors' accepted manuscript arising from this submission, in accordance with the grant's open access conditions.

The authors declare that they have no competing interests.
