# Supplementary material for: Biological subphenotypes in patients hospitalized with suspected infection in Thailand: a secondary analysis of a prospective observational study
Source: Lancet Reg Health Southeast Asia. 2025 Jan 30;33:100536. doi: 10.1016/j.lansea.2025.100536 (PMC11821389; doi:10.1016/j.lansea.2025.100536)
Supplement: Supplementary material [file mmc1.pdf]

Biological subphenotypes in patients hospitalized with suspected infection in Thailand: a secondary analysis of a prospective observational study.

Prapassorn Poolchanuan, Taylor D. Coston, Viriya Hantrakun, Parinya Chamnan, Gumphol Wongsuvan, Pavan K. Bhatraju, Narisara Chantratita, Direk Limmathurotsakul, T. Eoin West and Shelton W. Wright

Correspondence to:
Shelton Wright, MD MS
email:shelwi@uw.edu

Supplementary information

Contents (Page):

Supplementary Table 1: Infectious etiologies and presentation (2)
Supplementary Table 2: Data availability at enrollment (3)
Supplementary Table 3: Correlation matrix of variables (4)
Supplementary Table 4: Latent profile model fit characteristics (5)
Supplementary Table 5: Latent profile model sensitivity analysis without removal of biomarkers with high correlation (6)
Supplementary Table 6: Latent profile model sensitivity analysis with removal of correlated biomarkers (7)
Supplementary Table 7: Characteristics and outcome based on subphenotype for patients with modified SOFA≥2 (8)
Supplementary Table 8: Characteristics by subphenotype for infectious etiology and presentation (9)
Supplementary Table 9: Biomarker levels by subphenotype (10)
Supplementary Table 10: Parsimonious model characteristics in the derivation cohort (11)
Supplementary Table 11: Parsimonious model characteristics in the internal validation cohort (12-13)
Supplementary Table 12: ARDS subphenotypes (14)
Supplementary Figure 1: Study flow diagram (15)
Supplementary Figure 2: Discrimination of subphenotype assignment by the three-biomarker model (16)
Supplementary Figure 3: Distribution of ARDS and LBP subphenotypes (17)
Supplementary Methods and References (18)

Supplementary Table 1: Infectious etiologies and presentation

Variable, total available (%)	Cohort (N=585)
**Presenting syndrome**[a]	
Acute febrile illness	212 (36)
Suspected sepsis	197 (34)
Lower respiratory tract infection	166 (28)
Urinary tract infection	51 (9)
Skin or soft tissue infection	19 (3)
Gastrointestinal illness	99 (19)
Possible abscess	14 (2)
Other	36 (6)
**Blood stream infection**[b]	89 (15)
Gram negative organism	55 (61)
Burkholderia pseudomallei	17 (19)
Gram positive organism	27 (30)
Polymicrobial infection	4 (5)
Fungal infection	3 (3)
**Other infectious etiologies**[c]	
Non-bacteremic melioidosis	5 (1)
Malaria	12 (2)
Leptospirosis	21 (4)
Dengue	36 (6)
Scrub typhus	20 (3)

[a] Presenting syndrome based on primary diagnosis obtained from the medical record. Multiple diagnoses are possible.
[b] Blood stream infections were obtained from blood culture results at the time of admission and exclude species typically associated with contamination.
[c] Non-bacteremic melioidosis was determined by positive culture results from any culture including sputum, urine or other body fluid in the absence of a positive blood culture. Malaria, leptospirosis, dengue and scrub typhus etiologies were determined by the final diagnosis at the time of discharge.

Supplementary Table 2: Data availability at enrollment

Variable, total available (%)	Cohort (N=585)
**28-day mortality**	585 (100)
**Baseline risk factors**	
Sex	585 (100)
Age in years	585 (100)
Pre-existing condition data	585 (100)
Transfer status	585 (100)
Modified SOFA score	585 (100)
Venous lactate	585 (100)
**Laboratory variables**	
Platelets	584 (99.8)
WBC	584 (99.8)
Neutrophil %	582 (99.4)
Glucose	585 (100)
**Biomarkers**	
Ang-1	576 (98.4)
Ang-2	582 (99.4)
IL-1β	585 (100)
IL-6	585 (100)
IL-8	585 (100)
IL-10	585 (100)
TNF	585 (100)
sTNFR-1	585 (100)
sTREM-1	585 (100)
sFlt-1	582 (99.4)
PAI-1	585 (100)

Supplementary Table 3: Correlation matrix of variables[a]

	WBC	Plt	PMN	Gluc	IL-8	Ang-1	sTREM-1	Ang-2	sFlt-1	PAI-1	sTNFR-1	IL-10	IL-1β	IL-6	TNF
**WBC**	1.00														
**Plt**	0.26	1.00													
**PMN**	-0.49	-0.10	1.00												
**Gluc**	0.05	0.09	-0.02	1.00											
**IL-8**	-0.14	-0.39	-0.12	-0.10	1.00										
**Ang-1**	0.23	0.33	-0.20	0.16	0.02	1.00									
**sTREM-1**	0.34	-0.12	-0.41	0.01	0.44	0.16	1.00								
**Ang-2**	0.21	-0.27	-0.34	-0.04	0.48	0.06	0.62	1.00							
**sFlt-1**	-0.06	-0.39	-0.11	-0.07	0.48	-0.10	0.22	0.52	1.00						
**PAI-1**	-0.01	-0.22	-0.16	-0.05	0.62	0.21	0.43	0.48	0.48	1.00					
**sTNFR-1**	0.20	-0.28	-0.32	-0.03	0.52	0.01	0.82	0.67	0.31	0.45	1.00				
**IL-10**	-0.09	-0.52	-0.03	-0.09	0.63	-0.17	0.26	0.40	0.47	0.47	0.45	1.00			
**IL-1β**	0.12	-0.15	-0.24	-0.02	0.55	0.06	0.47	0.40	0.30	0.47	0.51	0.45	1.00		
**IL-6**	0.19	-0.27	-0.38	-0.08	0.64	0.04	0.57	0.61	0.45	0.56	0.61	0.60	0.67	1.00	
**TNF**	0.01	-0.44	-0.20	-0.08	0.61	-0.06	0.53	0.53	0.38	0.49	0.72	0.66	0.53	0.63	1.00

[a] Variable abbreviations: Plt= platelet count; Glu=glucose; Ang: angiopoietin; WBC: white blood cell count; IL: interleukin; PMN: neutrophil percentage; sTNFR-1: soluble tumor necrosis factor receptor-1; sTREM-1: soluble triggering receptor expressed by myeloid cells 1; TNF: tumor necrosis factor; PAI-1: plasminogen activator inhibitor 1.

Supplementary Table 4: Latent profile model fit characteristics

Number of profiles	Log likelihood	BIC[a]	Entropy[b]	Number of patients assigned to each profile				P value[c]
				1	2	3	4	
2	-10586	21309	0.964	497	88			<0.001
3	-10235	20655	0.880	305	227	53		<0.001
4	-10002	20238	0.894	55	205	273	52	0.17

[a] Bayesian information criterion (BIC) is a criterion for model selection with lower values suggesting model parsimony. BIC is sampled size adjusted.
[b] Entropy is an index of how well the classes are separated. It ranges from zero to one and values of 0.8 and up are generally considered a sign of a useful model.
[c] Vuong-Lo-Mendell-Rubin P value tests whether the number of classes provides improved model fit compared to a model using one fewer class

B. Probability of profile assignment in the three-profile model

	3-profile model		
	**LBP-1 (N=305)**	**LBP-2 (N=227)**	**LBP-3 (N=53)**
**Probability of assignment**			
**Median**	0.98	0.99	1.00
**IQR**	0.95-1.00	0.95-0.99	0.99-1.00
**10th-90th % range**	0.78-1.00	0.79-1.00	0.84-1.00

Supplementary Table 5: Latent profile model sensitivity analysis without removal of biomarkers with high correlation[a]

Number of profiles	Log likelihood	BIC[b]	Entropy[c]	Number of patients assigned to each profile				P value[d]
				1	2	3	4	
2	-11317	22780	0.964	488	97			<0.001
3	-10797	21793	0.908	247	281	57		<0.001
4	-10573	21396	0.910	230	55	242	58	0.49

B. Comparison of primary analysis and sensitivity analysis groups

	3-profile sensitivity analysis groups[e]		
**Primary analysis groups**	**Profile 1 (N=281)**	**Profile 2 (N=247)**	**Profile 3 (N=57)**
**LBP-1 (N=305)**	271/305 (89)	34/305 (11)	
**LBP-2 (N=227)**	10/227 (4)	213/227 (94)	4/227 (2)
**LBP-3 (N=53)**	0	0	53/53 (100)

[a] As it was removed in the primary model, sTNFR-1 was included in the sensitivity analysis.
[b] Bayesian information criterion (BIC) is a criterion for model selection with lower values suggesting model parsimony. BIC is sampled size adjusted.
[c] Entropy is an index of how well the classes are separated. It ranges from zero to one and values of 0.8 and up are generally considered a sign of a useful model.
[d] Vuong-Lo-Mendell-Rubin P value tests whether the number of classes provides improved model fit compared to a model using one fewer class
[e] A 3-profile group was selected as this profile had the lowest log likelihood, BIC and entropy while also maintaining minimal groups with <10% of the entire cohort.

Supplementary Table 6: Latent profile model sensitivity analysis with removal of correlated biomarkers[a]

Number of profiles	Log likelihood	BIC[b]	Entropy[c]	Number of patients assigned to each profile				P value[d]
				1	2			
2	-7823	15745	0.922	496	89			<0.001
3	-7622	15379	0.905	422	83	80		<0.001
4	-7526	15221	0.835	62	335	135	53	0.13

B. Comparison of primary analysis and sensitivity analysis groups

	3-profile sensitivity analysis groups[e]		
**Primary analysis groups**	**Profile 1 (N=422)**	**Profile 2 (N=80)**	**Profile 3 (N=83)**
**LBP-1 (N=305)**	254/305 (83)	51/305 (17)	0
**LBP-2 (N=227)**	168/227 (74)	29/227 (13)	30/227 (13)
**LBP-3 (N=53)**	0	0	53/53 (100)

[a] sTNFR-1, TNF, IL-6, IL-8 and sTREM-1 were removed for the sensitivity analysis.
[b] Bayesian information criterion (BIC) is a criterion for model selection with lower values suggesting model parsimony. BIC is sampled size adjusted.
[c] Entropy is an index of how well the classes are separated. It ranges from zero to one and values of 0.8 and up are generally considered a sign of a useful model.
[d] Vuong-Lo-Mendell-Rubin P value tests whether the number of classes provides improved model fit compared to a model using one fewer class
[e] A 3-profile group was selected as this profile had the lowest log likelihood, BIC and entropy while also maintaining minimal groups with <10% of the entire cohort.

Supplementary Table 7: Characteristics and outcome based on subphenotype for patients with modified SOFA≥2

Characteristics	LBP-1 (N=152)	LBP-2 (N=196)	LBP-3 (N=52)	P value
**Demographics**				
Age in years, median (IQR)	61 (39-76)	63 (51-74)	63 (47-73)	0·49
Female sex, N (%)	70 (46)	75 (38)	19 (37)	0·27
**Pre-existing conditions**				
Charlson Comorbidity Index, median (IQR)	3 (0-4)	3 (1-4)	2 (1-4)	0·45
Diabetes, N (%)	32 (21)	50 (26)	8 (15)	0·27
Liver disease, N (%)	5 (3)	5 (3)	3 (6)	0·49
Kidney disease, N (%)	19 (13)	36 (18)	9 (17)	0·31
Cardiovascular disease, N (%)	12 (8)	15 (8)	3 (6)	0·93
Lung disease, N (%)	13 (9)	10 (5)	3 (6)	0·43
Cancer, N (%)	7 (5)	4 (2)	1 (2)	0·39
HIV, N (%)	0 (0)	1 (1)	2 (4)	0·05
**Duration of symptoms**, median (IQR)	2 (1-4)	3 (1-4)	3 (1-3)	0·42
**Referred**, N (%)	93 (61)	126 (64)	37 (71)	0·43
Days to transfer, median (IQR)	0 (0-0)	0 (0-0)	0 (0-0)	0·92
**Lactate** (mmol/L)	1·6 (1·2-2·2)	2·1 (1·6-3·2)	6·4 (4·0-9·7)	<0·001
**Modified SOFA score**, median (IQR)	3 (2-5)	5 (3-7)	9 (7-12)	<0·001
**Critical care at enrollment**				
Mechanical ventilation, N (%)	26 (17)	45 (23)	29 (56)	<0·001
Receiving vasoactive medications, N (%)	31 (20)	56 (29)	31 (60)	<0·001
ICU admission, N (%)	6 (4)	14 (7)	15 (29)	<0·001
**28-day mortality**, N (%)	15 (10)	37 (19)	30 (58)	<0·001
Days to death[a], median (IQR)	6 (3-13)	8 (3-15)	2 (1-3)	<0·001

Supplementary Table 8: Characteristics by subphenotype for infectious etiology and presentation

Variable, total available (%)	LBP-1 (N=305)	LBP-2 (N=227)	LBP-3 (N=53)	P value
**Presenting syndrome**[a]				
Acute febrile illness	130 (43)	72 (32)	10 (19)	0.001
Suspected sepsis	60 (20)	99 (44)	38 (72)	<0.001
Lower respiratory tract infection	80 (26)	70 (31)	16 (30)	0.48
Urinary tract infection	26 (9)	23 (10)	2 (4)	0.33
Skin or soft tissue infection	4 (1)	14 (6)	1 (2)	0.01
Gastrointestinal illness	56 (18)	35 (15)	8 (15)	0.63
Suspected abscess	7 (2)	7 (3)	0	0.51
Suspected meningitis	6 (2)	2 (1)	2 (4)	0.30
**Blood stream infection**[b]	17 (6)	49 (22)	23 (43)	<0.001
Gram-negative organism	10 (3)	31 (14)	14 (26)	<0.001
Burkholderia pseudomallei	4 (1)	8 (4)	5 (9)	0.01
Gram-positive organism	6 (2)	14 (6)	7 (13)	0.001
Polymicrobial infection	0	3 (1)	1 (2)	0.1
Fungal infection	1 (0)	1 (0)	1 (2)	0.34
**Other infectious etiologies**[c]				
Non-bacteremic melioidosis	2 (1)	1 (0)	2 (4)	0.05
Malaria	4 (1)	7 (3)	1 (2)	0.36
Leptospirosis	6 (2)	12 (5)	3 (6)	0.09
Dengue	31 (10)	5 (2)	0	<0.001
Scrub typhus	10 (3)	10 (4)	0	0.32

[a] Presenting syndrome based on primary diagnosis obtained from the medical record. Multiple diagnoses are possible.
[b] Blood stream infections were obtained from blood culture results at the time of admission and exclude species typically associated with contamination.
[c] Non-bacteremic melioidosis was determined by positive culture results from any culture including sputum, urine or other body fluid in the absence of a positive blood culture. Malaria, leptospirosis, dengue and scrub typhus etiologies were determined by the final diagnosis at the time of discharge.

Supplementary Table 9: Biomarker levels by subphenotype

Variables (median, IQR)	LBP-1 (N=305)	LBP-2 (N=227)	LBP-3 (N=53)
**Laboratory variables**			
Platelets (#/mcL x1000)	217 (151-291)	181 (108-254)	55 (28-146)
WBC (#/mcL x1000)	11.2 (7.3-15.4)	13.6 (8.7-19.8)	13.8 (7.9-25.4)
Neutrophils (%)	78 (63-87)	84 (75-90)	88 (82-92)
Glucose (mg/dL)	124 (103-165)	144 (110-185)	108 (78-140)
**Biomarkers (pg/ml)**			
Ang-1	1957 (789-4011)	2019 (1107-3985)	2138 (1220-3522)
Ang-2 (x100)	21.3 (13.0-37.4)	65.0 (43.1-102.8)	176.5 (92.8-310.4)
IL-1β	0.3 (0.1-0.5)	0.8 (0.5-1.4)	7.0 (2.1-19.7)
IL-6 (x10)	0.9 (0.4-1.9)	5.9 (2.5-15.7)	1378.3 (168.0-1684.0)
IL-8	7.0 (4.0-12.2)	19.3 (10.5-41.5)	1326.6 (131.9-5731.5)
IL-10	1.6 (0.9-4.4)	7.9 (3.1-24.3)	205 (65.6-1383.3)
TNF	3.4 (2.3-5.2)	10.5 (5.6-17.6)	67.7 (31.4-120.5)
sTNFR-1 (x1000)	61 (41-100)	193 (132-334)	445 (328-668)
sTREM-1	272 (167-386)	681 (383-1027)	1504 (1179-2173)
sFlt-1 (x1000)	65 (28-102)	117 (68-211)	579 (284-987)
PAI-1 (x1000)	20 (12-33)	39 (22-75)	357 (178-580)

Supplementary Table 10: Parsimonious model characteristics in the derivation cohort

A. Derivation cohort: three-biomarker (IL-6 + sTREM-1 + Ang-2) model characteristics

IL-6 sTREM-1 Ang2	AUC	95% CI	Optimal cutoff[a]	Sensitivity	Specificity
**LBP-1**[b]	0.97	0.96-0.98	0.62	0.88	0.94
**LBP-2**[c]	0.78	0.73-0.83	0.33	0.86	0.64
**LBP-3**[d]	0.99	0.98-1.00	0.08	0.97	0.93

[a] Optimal cutoff to maximize Youden index; sensitivity and specificity based on cutoff
[b] LBP-1 model equation: logit(P) = 33.07395814 + log10 IL-6 * -3.64011754 + log10 sTREM-1 * -4.25564181 + log10 Ang-2 * -4.59074807
[c] LBP-2 model equation: logit(P) = -9.74193448 + log10 IL-6 * -0.15216504 + log10 sTREM-1 * 1.84104541 + log10 Ang-2 * 1.27496476
[d] LBP-3 model equation: logit(P) = -31.48705627 + log10 IL-6 * 2.86048493 + log10 sTREM-1 * 5.16238332 + log10 Ang-2 * 1.73852127

B. Derivation cohort: two-biomarker (IL-6 + sTREM-1) model characteristics

IL-6 sTREM-1	AUC	95% CI	Optimal cutoff[a]	Sensitivity	Specificity
**LBP-1**[b]	0.95	0.93-0.97	0.46	0.88	0.87
**LBP-2**[c]	0.76	0.71-0.81	0.31	0.88	0.57
**LBP-3**[d]	0.99	0.98-1.00	0.11	0.97	0.95

[a] Optimal cutoff to maximize Youden index; sensitivity and specificity based on cutoff
[b] LBP-1 model equation: logit(P) = 17.2407825 + log10 IL-6 * -3.34645562 + log10 sTREM-1 * -4.69801952
[c] LBP-2 model equation: logit(P) = -6.79336373 + log10 IL-6 * 0.04687595 + log10 sTREM-1 * 2.35790793
[d] LBP-3 model equation: logit(P) = -28.44042181 + log10 IL-6 * 2.99837501 + log10 sTREM-1 * 6.32495647

C. Derivation cohort: IL-6 model characteristics

IL-6	AUC	95% CI	Optimal cutoff[a]	Sensitivity	Specificity
**LBP-1**[b]	0.91	0.88-0.94	0.62	0.78	0.89
**LBP-2**[c]	0.76	0.71-0.81	0.36	0.88	0.64
**LBP-3**[d]	0.98	0.96-0.99	0.08	0.94	0.92

[a] Optimal cutoff to maximize Youden index; sensitivity and specificity based on cutoff
[b] LBP-1 model equation: logit(P) = 5.07475072 + log10 IL-6 * -3.53754916
[c] LBP-2 model equation: logit(P) = -1.27301321 + log10 IL-6 * 0.53243693
[d] LBP-3 model equation: logit(P) = -9.27521777 + log10 IL-6 * 2.87526055

Supplementary Table 11: Parsimonious model characteristics in the internal validation cohort

A. Confusion matrix and performance of the IL-6 + sTREM-1 + Ang-2 model in the internal validation cohort

		Actual		
	**Subphenotype**	**LBP-1 (N=127)**	**LBP-2 (N=88)**	**LBP-3 (N=19)**
**Assigned**	**LBP-1 (N=127)**	117	15	0
	**LBP-2 (N=88)**	10	69	3
	**LBP-3 (N=19)**	0	4	16

IL-6 sTREM-1 Ang2	AUC	95% CI	Optimal cutoff[a]	Sensitivity	Specificity	Accuracy[b]	F1[c]	Weighted F1[d]
**LBP-1**	0.96	0.94-0.98	0.62	0.87	0.93	0.86	0.88	0.89
**LBP-2**	0.77	0.71-0.83	0.33	0.85	0.59		0.89	
**LBP-3**	0.99	0.98-1.00	0.08	1.00	0.93		0.98	

[a] Optimal cutoff based on Youden index in the derivation cohort; sensitivity and specificity based on cutoff
[b] Accuracy determined based on true positives/all
[c] F1 score reflects the harmonic mean of precision (true positives/true positive + false positives) and recall (true positives/true positives + false negatives): (2 * precision * recall)/(precision + recall)
[d] Weighted F1 score: sample weighted mean of LBP F1 scores

B. Confusion matrix and performance of the IL-6 + sTREM-1 model in the internal validation cohort

		Actual		
	**Subphenotype**	**LBP-1 (N=127)**	**LBP-2 (N=88)**	**LBP-3 (N=19)**
**Assigned**	**LBP-1 (N=127)**	113	24	0
	**LBP-2 (N=88)**	14	61	3
	**LBP-3 (N=19)**	0	3	16

IL-6 sTREM-1	AUC	95% CI	Optimal cutoff[a]	Sensitivity	Specificity	Accuracy[b]	F1[c]	Weighted F1[d]
**LBP-1**	0.93	0.89-0.96	0.46	0.87	0.81	0.81	0.81	0.84
**LBP-2**	0.71	0.65-0.78	0.31	0.86	0.51		0.85	
**LBP-3**	0.99	0.99-1.00	0.11	1.00	0.93		0.99	

[a] Optimal cutoff based on Youden index in the derivation cohort; sensitivity and specificity based on cutoff
[b] Accuracy determined based on true positives/all
[c] F1 score reflects the harmonic mean of precision (true positives/true positive + false positives) and recall (true positives/true positives + false negatives): (2 * precision * recall)/(precision + recall)
[d] Weighted F1 score: sample weighted mean of LBP F1 scores

C. Confusion matrix and performance of the IL-6 model in the internal validation cohort

		Actual		
	Subphenotype	LBP-1 (N=127)	LBP-2 (N=88)	LBP-3 (N=19)
Assigned	LBP-1 (N=127)	113	31	0
	LBP-2 (N=88)	14	55	4
	LBP-3 (N=19)	0	2	15

IL-6	AUC	95% CI	Optimal cutoff[a]	Sensitivity	Specificity	Accuracy[b]	F1[c]	Weighted F1[d]
**LBP-1**	0.89	0.85-0.93	0.62	0.71	0.84	0.78	0.77	0.81
**LBP-2**	0.76	0.70-0.82	0.36	0.83	0.60		0.83	
**LBP-3**	0.99	0.98-1.00	0.08	1.00	0.94		0.99	

[a] Optimal cutoff based on Youden index in the derivation cohort; sensitivity and specificity based on cutoff
[b] Accuracy determined based on true positives/all
[c] F1 score reflects the harmonic mean of precision (true positives/true positive + false positives) and recall (true positives/true positives + false negatives): (2 * precision * recall)/(precision + recall)
[d] Weighted F1 score: sample weighted mean of LBP F1 scores

Supplementary Table 12: ARDS subphenotypes

A. Entire analysis cohort (N=585)

	**ARDS Hyperinflammatory (N=355/585, 61%)**	**ARDS Hypoinflammatory (N=230/585, 39%)**
**Latent Biological Profile**		
LBP-1, N (%)	95 (27)	210 (91)
LBP-2, N (%)	207 (58)	20 (9)
LBP-3, N (%)	53 (15)	0
**Baseline characteristics**		
Age, median (IQR)	63 (49-74)	52 (31-70)
Female sex, N (%)	155 (44)	128 (56)
Charlson comorbidity index, median (IQR)	3 (1-4)	1 (0-1)
Referred, N (%)	211 (59)	77 (34)
Modified SOFA score, median (IQR)	5 (3-7)	1 (0-3)
Lactate (mmol/L)	2.1 (1.6-3.6)	1.3 (1.1-1.9)
**28-day mortality**, N (%)	77 (22)	9 (4)

B. Patients with modified SOFA≥2 (N=400)

	**ARDS Hyperinflammatory (N=311/400, 78%)**	**ARDS Hypoinflammatory (N=89/400, 22%)**
**Latent Biological Profile**		
LBP-1, N (%)	76 (24)	76 (85)
LBP-2, N (%)	183 (59)	13 (15)
LBP-3, N (%)	52 (17)	0 (0)
**Baseline characteristics**		
Age, median (IQR)	63 (48-74)	63 (38-74)
Female sex, N (%)	127 (41)	37 (42)
Charlson comorbidity index, median (IQR)	3 (1-4)	3 (0-4)
Referred, N (%)	203 (65)	53 (60)
Modified SOFA score, median (IQR)	5 (3-8)	3 (2-4)
Lactate (mmol/L)	2.2 (1.6-3.8)	1.6 (1.2-2.3)
**28-day mortality**, N (%)	74 (24)	8 (9)

Supplementary Figure 1: Study flow diagram

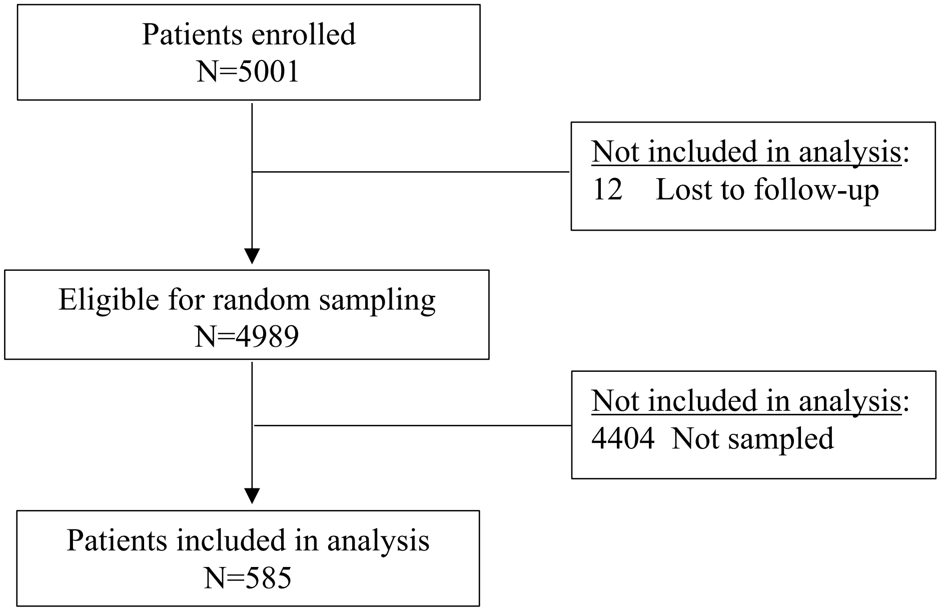

Supplementary Figure 2: Discrimination of subphenotype assignment by the three-biomarker model

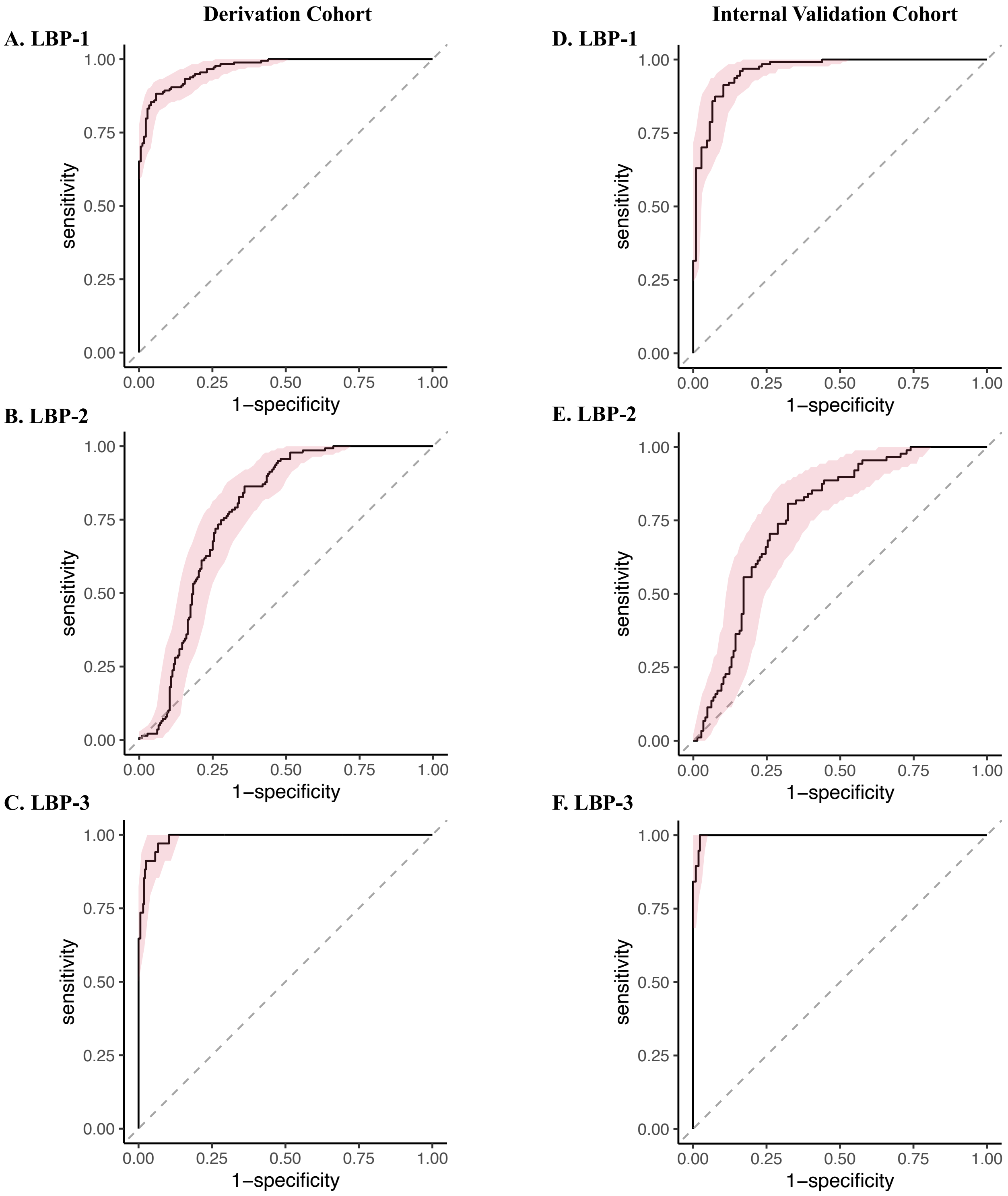

A-C) Area under the receiver operating characteristic curves (AUC) of a model containing IL-6, sTREM-1 and Ang-2 for assignment in the derivation cohort to LBP-1 (0.97, 95% CI 0.96-0.98), LBP-2 (0.78, 95% CI 0.73-0.83) and LBP-3 (0.99 (95% CI 0.98-1.00). **E-G)** AUC for assignment in the internal validation cohort to LBP-1 (0.96, 95% CI 0.94-0.98), LBP-2 (0.77, 95% CI 0.71-0.83) and LBP-3 (0.99 (95% CI 0.98-1.00).

Supplementary Figure 3: Distribution of ARDS and LBP subphenotypes

A. Entire analysis cohort (N=585)

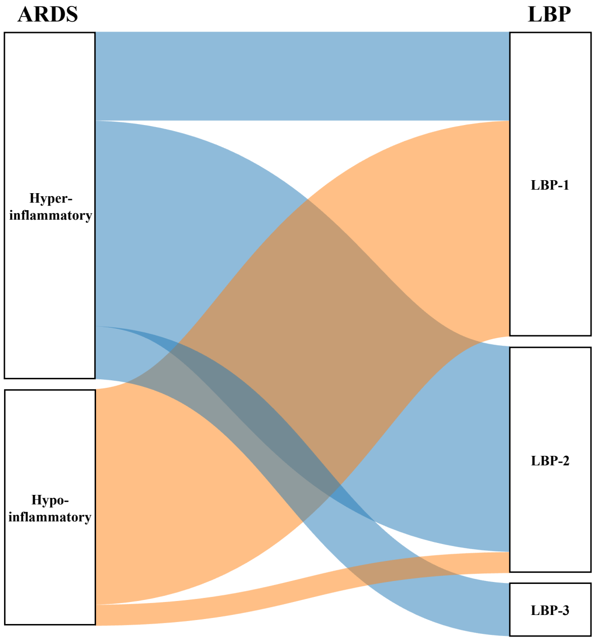

B. Patients with modified SOFA≥2 (N=400)

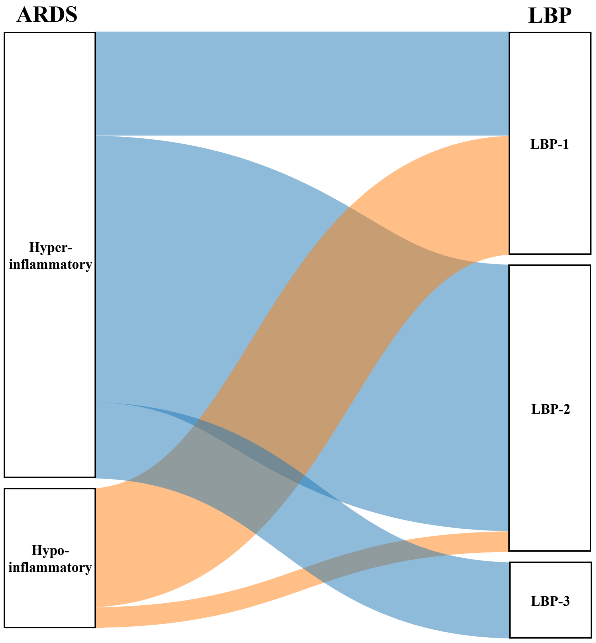

Alluvial plots showing concordance of ARDS and LBP subphenotypes in **A)** the entire cohort and **B)** restricting the cohort to patients with modified SOFA ≥2.

Supplementary methods

Parsimonious subphenotype classification model development

A Youden index threshold representing optimal discrimination by maximizing sensitivity and specificity was determined for classification of each LBP. Subsequently, the sensitivity and specificity for LBP assignment at the index threshold was calculated. In the internal validation cohort, model performance was also assessed by generating a confusion matrix, F1 scores for each subphenotype as well as calculating classification accuracy and a weighted F1 score for each model.[1]

ARDS subphenotypes

ARDS subphenotypes were calculated using the following published formula as bicarbonate was not available (y=-18.4764+[1.3367 ln ([sTNFR-1]+1)]+[1.3013 ([IL-8]+1)]+2.3439[vasopressor (yes/no)]).[2] The probability was calculated as (probability= $(e^y)/(1+e^y)$). If the probability was >0.5, the patient was classified in the "hyperinflammatory" subphenotype; otherwise, patients were classified in the "hypoinflammatory" subphenotype.

Supplementary references

1 Varoquaux G, Colliot O. Evaluating Machine Learning Models and Their Diagnostic Value. In: Colliot O, ed. Machine Learning for Brain Disorders. New York, NY: Humana, 2023: 601–30.

2 Sinha P, Delucchi KL, McAuley DF, O'Kane CM, Matthay MA, Calfee CS. Development and validation of parsimonious algorithms to classify acute respiratory distress syndrome phenotypes: a secondary analysis of randomised controlled trials. *Lancet Respir Med* 2020; **8**: 247–57.
